# Association between the C-reactive protein-triglyceride glucose index and erectile dysfunction in US males: results from NHANES 2001–2004

**DOI:** 10.1038/s41443-024-00945-z

**Published:** 2024-07-04

**Authors:** Yangyang Mei, Yangmeina Li, Bo Zhang, Renfang Xu, Xingliang Feng

**Affiliations:** 1 Department of Urology, Jiangyin People’s Hospital Affiliated to Nantong University, Jiangyin, Jiangsu China; 2 Department of Otolaryngology, The Third Affiliated Hospital of Soochow University, Changzhou, Jiangsu China; 3 Department of Urology, The First People’s Hospital of Changzhou, Changzhou, Jiangsu China; 4 Department of Urology, The Third Affiliated Hospital of Soochow University, Changzhou, Jiangsu China

**Keywords:** Diagnostic markers, Risk factors

## Abstract

The C-reactive protein-triglyceride glucose index (CTI) is emerging as a novel indicator for comprehensively assessing the severity of both inflammation and insulin resistance. However, the association between CTI and erectile dysfunction (ED) remains largely unexplored. Participant data for this study were sourced from NHANES 2001–2004, with exclusion criteria applied to those lacking information on clinical variables. The CTI was defined as 0.412*Ln (CRP) + ln [T.G. (mg/dL) × FPG (mg/dL)/2]. Weighted univariable and multivariable logistic regression models were utilized to examine the correlation between the CTI and ED, assessing the CTI as both a continuous and categorical variable (quartile). Moreover, subgroup analyses were conducted to pinpoint sensitive populations, and interaction analysis was performed to validate the findings. A total of 1502 participants were included in the final analysis, encompassing 302 with ED and 1200 without ED. After adjusting for potential confounders, the CTI was positively associated with ED incidence (OR = 1.56, 95% CI: 1.27–1.90, *P* = 0.002). The fourth quartile of the CTI significantly increased the incidence of ED (OR = 2.69, 95% CI: 1.07–6.74, *P* = 0.04), and the lowest quartile of CTI was used as the reference. The dose-response curve revealed a positive linear relationship between the CTI and the incidence of ED. Subgroup analysis confirmed the consistent positive relationship between the CTI and ED. The interaction test indicated no significant impact on this association. Finally, a sensitivity analysis was performed to verify the significant positive correlation between the CTI and severe ED (OR = 1.44, 95% CI: 1.19–1.76, *P* = 0.004). Our national data indicate that a greater CTI is positively linked to an increased risk of ED in US men, suggesting its potential for use in clinical practice for ED prevention or early intervention. Additional large-scale prospective studies are warranted to substantiate the causative relationship between CTI and ED.

## Introduction

Erectile dysfunction (ED) is characterized by the chronic and recurring incapacity to obtain or maintain a sufficient erection for satisfactory intercourse [1]. It is a globally prevalent ailment that primarily affects men aged over 40 years old [2]. According to the Massachusetts Male Aging Study, it’s reported that mild to moderate ED affects 52% of men aged between 40 and 70 years old, with the prevalence of severe ED rising from 5% to 15% with advancing age [3]. In contrast, the prevalence of ED is less than 10% in men under age 40, which is considerably lower than the prevalence observed in men aged over 40 [4]. Furthermore, the global incidence of ED is expected to reach approximately 322 million by 2025 [5]. While not life-threatening, ED significantly impairs harmonious sexual life, thereby reducing overall quality of life [6].

Penile erection is a complex neurovascular phenomenon that depends on the integrity of neurological, vascular, endocrinological, psychological, and relational factors [7]. Notably, vascular factors exert more significant impacts on ED due to the particular vascular network of the penis [8]. Thus, in addition to its association with age, ED is often linked to obesity, physical inactivity, diabetes, hypertension, dyslipidemia, and cardiovascular diseases (CVD) [9–11]. Among the underlying mechanisms of these risk factors, insulin resistance (IR) and inflammation play decisive roles in the pathology of ED, disrupting the endothelial functions and structures [12, 13]. Former IR could lead to an excessive consumption of nitric oxide (NO) in tissues, resulting in a subsequent reduction in NO synthesis and release [14]. NO stimulates soluble guanylate cyclase, leading to elevated cyclic guanosine monophosphate (cGMP) levels in smooth muscle cells (SMCs), thereby playing a pivotal role in penile erection [15]. Such inflammation may inhibit the expression of the endothelial nitric oxide synthase (eNOS) gene in endothelial cells (ECs), ultimately resulting in endothelial dysfunction [16]. More importantly, IR and inflammation can mutually reinforce each other, creating a vicious cycle that progressively exacerbates damage to ECs and SMCs, which are critical cellular structures for the proper functioning of the penis [17, 18]. Previous studies have demonstrated a negative association between ED and the triglyceride glucose (TyG) index, a sensitive marker for IR [19, 20]. IR is a condition in which the production of insulin in normal amounts cannot optimally trigger the transfer of glucose from the blood to peripheral tissues, thereby leading to disorders in glucose and lipid metabolism [21]. The TyG index is calculated using fasting blood glucose and fasting triglycerides levels and simultaneously reflects abnormalities in glucose and lipid metabolism within the body. Consequently, compared with the hyperinsulinemic-euglycemic clamp (HEC) gold standard method, this approach demonstrated an excellent association with the diagnosis of IR [22]. Furthermore, studies have revealed that patients with ED often exhibit elevated levels of inflammatory markers such as C-reactive protein (CRP), interleukins, and other systemic inflammatory indicators [23, 24]. However, to date, no studies have investigated the combined effects of IR and inflammation on the onset of ED, which may stem from a lack of relevant indicators capable of comprehensively assessing both factors.

Ruan et al. introduced a novel metric termed the “C-reactive protein-triglyceride glucose index (CTI)” to provide a comprehensive assessment of both inflammatory status and IR [25]. Research has shown the potential of CTI in predicting survival among both Chinese and American cancer populations, underscoring its significance in enhancing the risk stratification for incident cancer mortality [25, 26]. The CTI provides a foundation for our exploration of the combined effects of IR and inflammation on the onset of ED. Hence, our objective was to investigate the correlation between CTI and ED using a large cohort from the National Health and Nutrition Examination Survey (NHANES), providing robust evidence regarding this association.

## Materials and methods

### Data source and study population

Data were extracted from the NHANES database, a national project by the Centers for Disease Control and Prevention’s National Center for Health Statistics (NCHS), aimed at assessing the health and nutritional status of the American population. The survey combines interviews and physical examinations by experienced medical personnel to collect data on sociodemographic traits, physiological examinations, nutritional status indicators, laboratory tests, and overall health. Using a sophisticated multistage probability sampling method, the survey collects a representative sample of noninstitutionalized civilian residents across the US. All NHANES protocols were thoroughly reviewed and approved by the NCHS Research Ethics Review Committee (NCHS IRB/ERB Protocol No. #98-12), and all participants provided written consent before taking part in the research.

**Fig. 1:**
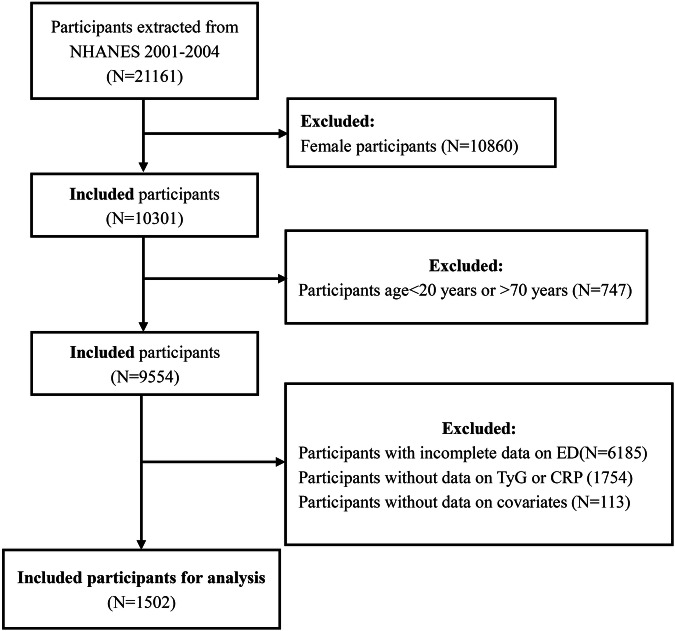
The flow chart of selection process for participants of NHANES 2001–2004.

We extracted two NHANES survey cycles data (2001–2002, 2003–2004) for analysis because only these two survey cycles included investigations into ED. Initially, 21,161 individuals were included in the survey which was conducted from 2001 to 2004. The following criteria were used to exclude individuals from the study population: (1) female participants (*N* = 10,860); (2) lacking ED information (*N* = 6185); (3) aged <20 years or >70 years (*N* = 747); (4) lacking TyG or CRP information (*N* = 1754); (5) lacking potential covariate information (*N* = 113). Ultimately, the study comprised 1502 participants, with 302 diagnosed with ED and 1200 without. Figure 1 illustrates the detailed sample selection process and criteria.

### Measurement of ED

According to the NHANES data, the presence of ED was primarily ascertained through a self-report format. Specifically, ED was assessed through responses to the following questionnaire: “How would you describe your ability to achieve and maintain sufficient erections for satisfactory intercourse?”. This query has been validated as an accurate method for identifying men with a medical diagnosis of ED [27]. Potential responses included “never able to get and maintain an erection”, “sometimes able to get and maintain an erection”, “usually able to get and maintain an erection”, and “always or almost always able to get and maintain an erection”. Participants who responded with ‘never able to get and maintain an erection’ or ‘sometimes able to get and maintain an erection’ were categorized as having ED, whereas the others were classified as not having ED. In the sensitivity analysis, a more stringent definition of ED was applied: Participants who answered “never able to get and maintain an erection” were identified as having ED.

### Measurement of the CTI

The CTI [25] was defined as 0.412*Ln (CRP) + TyG, and the TyG was defined as Ln [fasting triglyceride (TG) (mg/dL) × fasting glucose (FPG) (mg/dL)/2] [28]. Blood samples were collected in the morning after 8.5 h of fasting and sent to NCHS-certified laboratories for processing. The Roche Modular P and Roche Cobas 6000 chemistry analyzers were employed to determine serum triglyceride levels, and the oxygen rate method on the Beckman DxC800 was used to measure FPG. Moreover, CRP levels were quantified using latex-enhanced nephelometry on a Behring Nephelometer. Detailed information about the laboratory examinations can be found on the NHANES official website. Typically, a higher CTI signifies more severe inflammation and IR.

### Measurement of covariates

According to available studies, the covariates potentially influencing the association between ED and CTI include age, race, educational level, marital status, the family poverty income ratio (PIR), body mass index (BMI), smoking status, alcohol intake, physical activity (vigorous/moderate), hypertension, diabetes, hyperlipidemia, and CVD [19, 24]. Race/ethnic background was divided into 5 categories: Mexican American, non-Hispanic White, non-Hispanic Black, other Hispanic, and other races. Education level was segmented into below high school, high school, and above high school, while marital status was classified as living alone or being married/living with a partner. The PIR, indicative of economic status, was categorized as ≤1.3, 1.3–3.5, and >3.5. Alcohol intake was classified as either no (<1 drink per week) or yes (≥1 drink per week). Men who had never smoked more than 100 cigarettes throughout their lives were classified as nonsmokers. Those who had smoked more than 100 cigarettes throughout their lives but were not smoking at the time of the interview were categorized as former smokers. The remaining men were identified as current smokers. Physical activity status was determined based on responses to whether individuals engaged in moderate or vigorous activity in the past month. Men were classified as having a diabetic diagnosis if they had previously been diagnosed with a diabetes condition or a fasting plasma glucose level equal to or exceeding 126 mg/dL. Men who disclosed a prior diagnosis of angina, heart attack, or coronary heart disease were categorized as having CVD. Blood pressure measurement data were collected from participants with four readings of systolic and diastolic measurements on two separate occasions. Hypertension was identified by the average of these four measurements (≥140/90 mmHg), a previous diagnosis of high blood pressure, or self-reported usage of antihypertensive medication. Hypercholesterolemia was characterized by a total cholesterol level equal to or exceeding 240 mg/dL, a previous diagnosis of “high cholesterol”, or self-reported usage of cholesterol-lowering medication.

### Statistical analysis

To mitigate the impact of the complex multistage sampling design used in the NHANES, weighted analysis was performed using appropriate sample weights as outlined in the NHANES guidelines to enhance data accuracy. To derive prevalence estimates representative of the U.S. population, mobile examination center (MEC) weights were applied. Due to the integration of data from two cross-sectional studies, the final weights were determined by multiplying the original MEC weights by 0.5 [29]. The weighted means ± SEs for continuous variables and weighted percentages (%) for categorical variables are presented to illustrate demographic traits. Survey-weighted linear regression was employed to assess baseline characteristics of ED status for continuous variables, while a survey-weighted chi-square test was used for categorical variables. Weighted univariate (crude model) and weighted multivariate logistic regression analyses (adjusted models 1 and 2) were utilized to assess the relationship between the CTI and ED. Model 1 was adjusted for age, race/ethnic background, PIR, marital status, and educational level. Model 2 was adjusted for BMI, smoking status, alcohol intake, hypertension, DM, CVD, hypercholesterolemia, moderate physical activity, and vigorous physical activity on the basis of Model 1. The logistic regression outcomes are depicted using the odds ratio (OR) and its corresponding 95% confidence interval (CI) to measure the strength of the association.

To assess robustness, the CTI was converted from a continuous variable to a categorical variable based on its quartiles. A linear trend test was then performed using the four CTI quartiles as continuous measures. To establish whether the CTI showed a linear relationship with ED, smooth curve fitting and generalized additive modeling were employed under Model 2. Additionally, subgroup analyses were conducted to explore the relationship between the CTI and ED within various subgroups. As part of the sensitivity analysis, ED was defined more strictly, and all analyses were repeated to further validate our findings. All statistical analyses were performed using the software packages R (http://www.R-project.org; The R Foundation) and Empower (www.empowerstats.com). A two-tailed *P*-value < 0.05 was considered to indicate statistical significance.

## Results

### Baseline characteristics of the study population

Ultimately, the study enrolled 1502 individuals in total; Table 1 presents the weighted fundamental attributes of the study population, grouped by the presence or absence of ED. The CTI of ED participant was significantly greater than that of non-ED participants (8.53 ± 0.05 vs. 7.94 ± 0.03, *P* < 0.0001). Compared with participants without ED, men with ED were more advanced in age, had a BMI greater than 30 kg/m^2^, were less educated, were more likely to cohabit with a partner, be a former smoker, be an alcohol user, and not be involving in vigorous activities (all *P* < 0.05). Additionally, a relatively higher proportion of participants in the ED group displayed comorbidities (such as hypertension, DM, CVD, and hypercholesterolemia) than those without ED.

**Table 1:** Baseline characteristics of the study population based on presence of erectile dysfunction, weighted.

Characteristics	Total participants	History of erectile dysfunction (ED)		*P*-value
		No	Yes	
Number, *n*	1502	1200	302	
Age, year	42.45 ± 0.50	40.27 ± 0.43	53.95 ± 0.77	<0.0001
BMI, kg/m^2^	28.35 ± 0.15	28.03 ± 0.17	30.04 ± 0.55	0.0026
CRP, mg/dl	0.35 ± 0.03	0.33 ± 0.03	0.43 ± 0.04	0.0127
Fasting triglyceride, mg/dl	167.41 ± 5.53	158.28 ± 4.74	215.76 ± 20.22	0.0073
Fasting glucose, mg/dl	103.58 ± 0.73	100.52 ± 0.72	119.78 ± 2.92	<0.0001
TyG	8.81 ± 0.02	8.75 ± 0.02	9.14 ± 0.04	<0.0001
CTI	8.03 ± 0.03	7.94 ± 0.03	8.53 ± 0.05	<0.0001
Age, %				<0.0001
<50 y	68.10 ± 0.03	75.73 ± 1.76	27.74 ± 3.23	
≥50 y	31.90 ± 0.03	24.27 ± 1.76	72.26 ± 3.23	
BMI, %				0.0046
<25 kg/m^2^	27.49 ± 0.02	29.10 ± 1.27	19.01 ± 2.95	
≥25 kg/m^2^ and <30 kg/m^2^	42.45 ± 0.03	42.80 ± 1.92	40.60 ± 2.81	
≥30 kg/m^2^	30.06 ± 0.02	28.10 ± 1.77	40.39 ± 3.73	
Race, %				0.5619
Mexican American	8.69 ± 0.01	8.80 ± 1.22	8.13 ± 1.90	
Non-Hispanic White	73.82 ± 0.05	73.32 ± 2.74	76.48 ± 3.55	
Non-Hispanic Black	9.59 ± 0.01	9.86 ± 1.24	8.15 ± 1.41	
Other Hispanic	3.68 ± 0.01	3.53 ± 1.03	4.47 ± 2.58	
Other races	4.22 ± 0.01	4.49 ± 0.74	2.77 ± 1.17	
Educational level, %				0.0043
Below high school	15.81 ± 0.01	14.19 ± 0.91	24.39 ± 3.23	
High school	28.66 ± 0.02	29.47 ± 1.54	24.34 ± 2.92	
Above high school	55.53 ± 0.03	56.34 ± 1.43	51.27 ± 4.24	
Marital status, %				0.0016
Living alone	29.85 ± 0.02	31.54 ± 1.98	20.90 ± 2.85	
Married or living with a partner	70.15 ± 0.04	68.46 ± 1.98	79.10 ± 2.85	
PIR, %				0.6483
PIR ≤ 1.3	15.51 ± 0.01	15.23 ± 1.20	16.99 ± 2.37	
1.3 < PIR ≤ 3.5	35.94 ± 0.02	35.68 ± 1.75	37.35 ± 3.88	
PIR > 3.5	48.55 ± 0.03	49.09 ± 1.86	45.66 ± 4.31	
Alcohol intake, %				<0.001
No	22.57 ± 0.03	20.25 ± 2.34	34.83 ± 3.88	
Yes	77.43 ± 0.04	79.75 ± 2.34	65.17 ± 3.88	
Smoking, %				<0.0001
Never	43.35 ± 0.02	45.98 ± 2.15	29.41 ± 3.74	
Former	27.06 ± 0.02	24.48 ± 1.53	40.69 ± 3.60	
Now	29.59 ± 0.02	29.53 ± 1.41	29.90 ± 3.27	
Vigorous activity				<0.0001
No	59.74 ± 0.04	56.59 ± 1.78	76.37 ± 3.04	
Yes	40.26 ± 0.02	43.41 ± 1.78	23.63 ± 3.04	
Moderate activity				0.0776
No	43.77 ± 0.03	42.70 ± 1.56	49.46 ± 3.94	
Yes	56.23 ± 0.03	57.30 ± 1.56	50.54 ± 3.94	
History of diabetes, %				<0.0001
No	90.00 ± 0.04	94.21 ± 0.69	67.70 ± 3.11	
Yes	10.00 ± 0.01	5.79 ± 0.69	32.30 ± 3.11	
History of CVD, %				<0.0001
No	96.26 ± 0.05	97.84 ± 0.47	87.94 ± 2.72	
Yes	3.74 ± 0.01	2.16 ± 0.47	12.06 ± 2.72	
History of hypertension, %				<0.0001
No	67.42 ± 0.03	71.49 ± 1.50	45.89 ± 3.32	
Yes	32.58 ± 0.02	28.51 ± 1.50	54.11 ± 3.32	
History of hypercholesterolemia %				0.0015
No	26.36 ± 0.02	27.95 ± 1.44	17.98 ± 2.36	
Yes	73.64 ± 0.04	72.05 ± 1.44	82.02 ± 2.36	

Statistical tests: Continuous variables were presented as mean ± standard error (x̅ ± SE), and the categorical variables were expressed as percentages (%) ± SE. The survey-weighted linear regression and weighted chi-square test were used to compare the differences between the two groups for continuous variables and categorical variables.

*ED* erectile dysfunction, *BMI* body mass index, *CRP* C-reactive protein, *TyG* triglyceride glucose index, *CTI* C-reactive protein-triglyceride glucose index, *PIR* poverty income ratio, *CVD* cardiovascular disease.

### Association between the CTI and ED

**Fig. 2:**
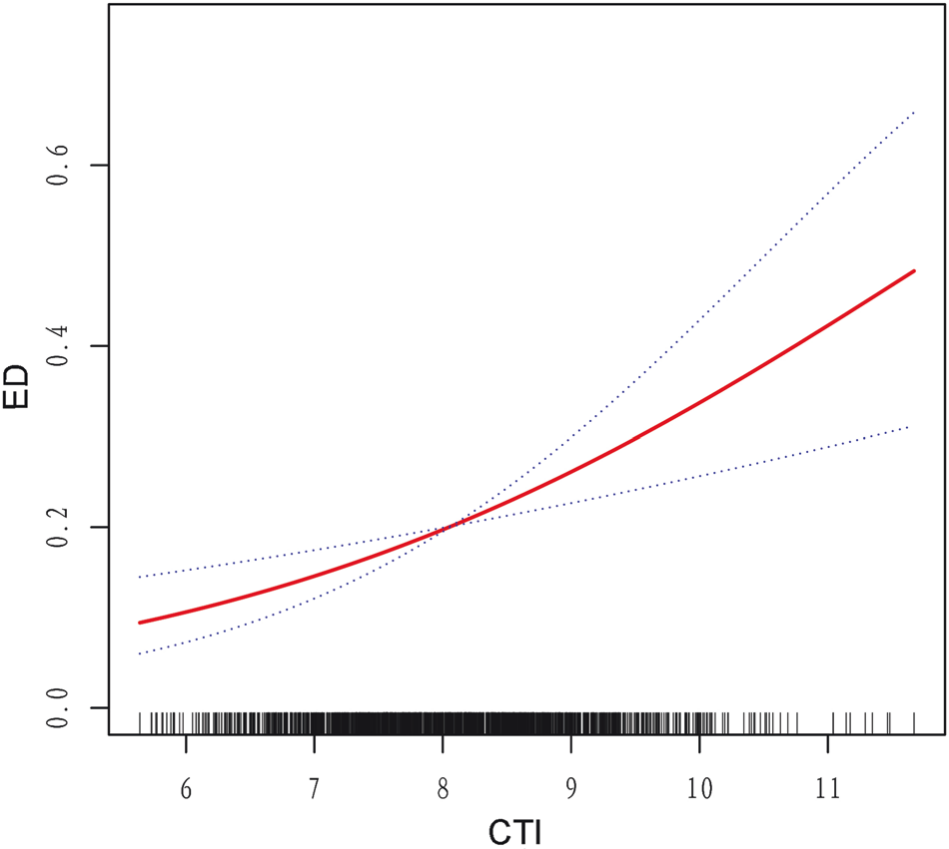
Density dose-response relationship between CTI with ED prevalence. The area between the upper and lower dashed lines is represented as 95% CI. The red line indicates that the positive linear association between CTI and ED is proven by generalized additive model. Adjusted for all confounders in model 2 included age, race, PIR, marital status, educational level, BMI, smoking status, alcohol intake, hypertension, DM, CVD, hypercholesterolemia, moderate physical activity, and vigorous physical activity.

Various regression analyses, incorporating different adjustments to control for confounding factors, revealed a consistent positive association between CTI and ED incidence among all models. Table 2 depicts the detailed results of the analysis. According to Model 2 with full adjustments, each incremental increase in the CTI was significantly associated with a 56% rise in the risk of ED (OR = 1.56, 95% CI: 1.27–1.90, *P* = 0.002). When categorizing the CTI into four quartiles (Q1: 5.64–7.42, Q2: 7.42–8.06, Q3: 8.06–8.65, Q4: 8.65–11.67), logistic regression revealed that participants in the highest quartile (Q4) had a significant 1.69-fold greater incidence of ED than did those in the lowest quartile (Q1). (OR = 2.69, 95% CI: 1.07–6.74, *P* = 0.04). This observation was further substantiated by a significant *P*-value for the trend (*P* = 0.015), illustrating a robust association between the CTI and ED incidence. Our findings showed a linear positive association between the CTI and ED incidence through generalized additive modeling and smoothed curve fitting in the fully adjusted Model 2, which was comparable to the outcomes of logistic regression analysis (Fig. 2).

**Table 2:** Weighted multivariable logistic regression for the association between CTI and ED prevalence.

Exposure	Crude model		Adjusted model 1		Adjusted model 2	
	OR (95%CI)	*P*-value	OR (95% CI)	*P*-value	OR (95% CI)	*P*-value
CTI (continuous)	2.01 (1.78, 2.26)	<0.0001	1.79 (1.56, 2.05)	<0.0001	1.56 (1.27, 1.90)	0.002
CTI (Quartiles)						
Q1 (5.64–7.42)	Ref.		Ref.		Ref.	
Q2 (7.42–8.06)	1.78 (1.03, 3.06)	0.04	1.45 (0.83, 2.54)	0.18	1.48 (0.60, 3.66)	0.29
Q3 (8.06–8.65)	2.33 (1.30, 4.17)	0.01	1.82 (0.96, 3.45)	0.07	1.64 (0.58, 4.65)	0.26
Q4 (8.65–11.67)	5.07 (3.47, 7.41)	<0.0001	3.59 (2.39, 5.38)	<0.0001	2.69 (1.07, 6.74)	0.04
*P* for trend	<0.0001		<0.0001		0.015	

Statistical tests: Crude Model: no covariates were adjusted.

Model 1: age, race, PIR, marital status, and educational level were adjusted.

Model 2: Model 1 + BMI, smoking status, alcohol intake, hypertension, DM, CVD, hypercholesterolemia, moderate physical activity, and vigorous physical activity were adjusted.

*CTI* C-reactive protein-triglyceride glucose index, *ED* Erectile dysfunction, *OR* odds ratio, *CI* confidence interval, *Q1–Q4* quartile 1 to quartile 4, *CVD* cardiovascular disease, *DM* diabetes mellitus, *BMI* body mass index.

### Subgroup analysis

**Fig. 3:**
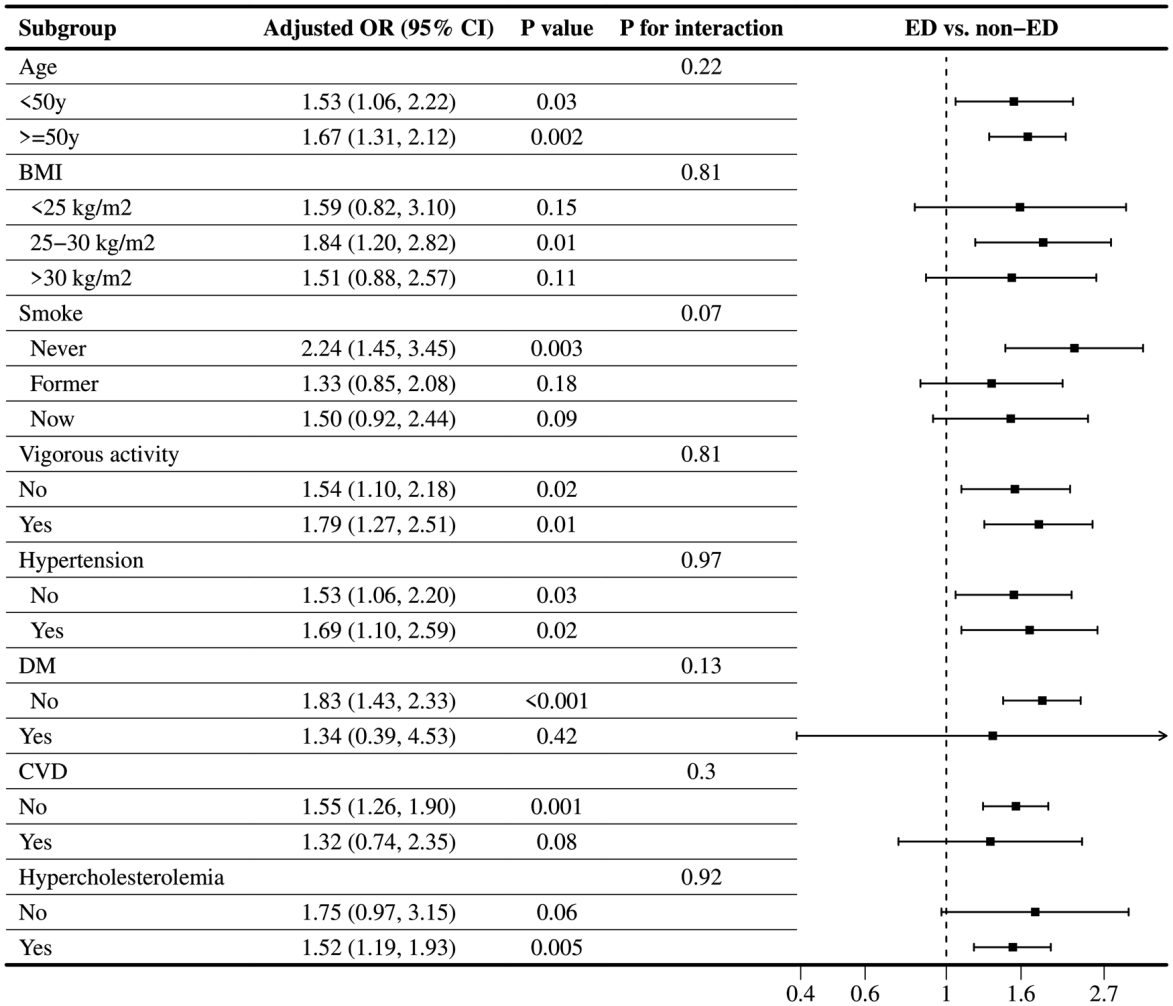
Subgroup analysis for the relationship between CTI and ED with CTI as continuous variable. All factors including age, BMI, smoking status, vigorous activity, and a history of hypertension, DM, CVD, and hypercholesterolemia had no impact on the independent positive association between CTI and ED. All subgroups were adjusted for age, race, PIR, marital status, educational level, BMI, smoking status, alcohol intake, hypertension, DM, CVD, hypercholesterolemia, moderate physical activity, and vigorous physical activity, except the stratification factor itself.

Stratified analyses were performed to further explore the stability of the association between the CTI and ED in different subgroups. As illustrated in Table 3, utilizing the CTI as a categorical variable, all subgroup analyses were conducted on age, BMI, smoking status, vigorous activity, and a history of hypertension, DM, CVD, and hypercholesterolemia. The results suggested that none of the stratification variables had an impact on the positive correlation between the CTI and ED (all *P*-values for interactions >0.05). The results of subgroup analyses are also presented in Fig. 3, where the CTI was considered as a continuous variable. Similar to the aforementioned findings, no significant influence across all subgroups was detected on the positive correlation between the CTI and ED incidence. Notably, some stratifications showed statistically stable significance for the positive relationship. Participants under 50 years of age displayed a 53% greater risk of ED for each unit increase in the CTI (OR = 1.53, 95% CI: 1.06–2.22, *P* = 0.03), while those aged 50 and above exhibited a 67% greater probability of ED (OR = 1.67, 95% CI: 1.31–2.12, *P* = 0.002).

**Table 3:** Subgroup analysis for the relationship CTI quartiles and ED in fully adjusted model 2 except the stratification factor itself, weighted.

Subgroup	Q1	Q2	Q3	Q4	*P* for trend	*P* for interaction
	Ref.	OR (95%CI)	OR (95%CI)	OR (95%CI)		
Age						0.83
<50 y	Ref.	1.65 (0.52, 5.27)	1.54 (0.39, 6.00)	3.26 (0.88,12.13)	0.07	
≥50 y	Ref.	1.30 (0.40, 4.21)	1.79 (0.55, 5.76)	2.54 (0.96, 6.67)	0.02	
BMI						0.78
<25 kg/m^2^	Ref.	1.27 (0.33, 4.97)	1.76 (0.34, 9.20)	1.78 (0.32, 9.85)	0.41	
25~30 kg/m^2^	Ref.	2.67 (0.85, 8.41)	2.46 (0.50, 12.02)	6.21 (1.59, 24.22)	0.01	
>30 kg/m^2^	Ref.	0.65 (0.09, 4.68)	0.95 (0.13, 6.87)	1.27 (0.22, 7.37)	0.33	
Smoke						0.07
Never	Ref.	4.50 (0.84, 24.17)	10.73 (2.62, 43.92)	12.73 (2.59, 62.54)	0.003	
Former	Ref.	0.72 (0.22, 2.40)	0.42 (0.11, 1.63)	1.27 (0.34, 4.72)	0.49	
Now	Ref.	1.65 (0.58, 4.68)	1.38 (0.32, 5.89)	2.37 (0.55, 10.28)	0.26	
Vigorous activity						0.09
No	Ref.	0.80 (0.31, 2.07)	1.21 (0.34, 4.26)	2.10 (0.61, 7.26)	0.06	
Yes	Ref.	5.34 (1.33, 21.49)	3.16 (0.66, 15.07)	4.99 (1.03, 24.02)	0.09	
Hypertension						0.91
No	Ref.	1.55 (0.50, 4.77)	1.44 (0.41, 5.07)	2.73 (0.78, 9.55)	0.08	
Yes	Ref.	1.75 (0.38, 8.04)	2.05 (0.60, 6.96)	2.98 (0.71,12.61)	0.06	
DM						0.09
No	Ref.	1.81 (0.72, 4.53)	1.82 (0.63, 5.27)	3.79 (1.47, 9.80)	0.01	
Yes	Ref.	0.03 (0.00, 0.39)	0.10 (0.01, 0.80)	0.11 (0.01, 1.13)	0.77	
CVD						0.73
No	Ref.	1.44 (0.61, 3.36)	1.58 (0.56, 4.46)	2.69 (1.10, 6.62)	0.02	
Yes	Ref.	1.46 (0.12, 1.72)	3.66 (0.14, 6.91)	2.303 (0.08, 6.71)	0.46	
Hypercholesterolemia						0.47
No	Ref.	2.12 (0.52, 8.57)	1.51 (0.45, 5.08)	2.05 (0.44, 9.57)	0.17	
Yes	Ref.	1.22 (0.54, 2.76)	1.62 (0.56, 4.68)	2.43 (0.94, 6.24)	0.02	

Statistical tests: Model 2 controlling for age, race, PIR education, marital status, BMI, alcohol use, smoking, diabetes, hypertension, CVD, hypercholesterolemia, moderate physical activity, and vigorous physical activity were adjusted.

*CTI* C-reactive protein-triglyceride glucose index, *ED* Erectile dysfunction, *OR* odds ratio, *CI* confidence interval, *Q1–Q4* quartile 1 to quartile 4, *CVD* cardiovascular disease, *DM* diabetes mellitus, *BMI* body mass index, *PIR* poverty income ratio.

### Sensitivity analysis

**Fig. 4:**
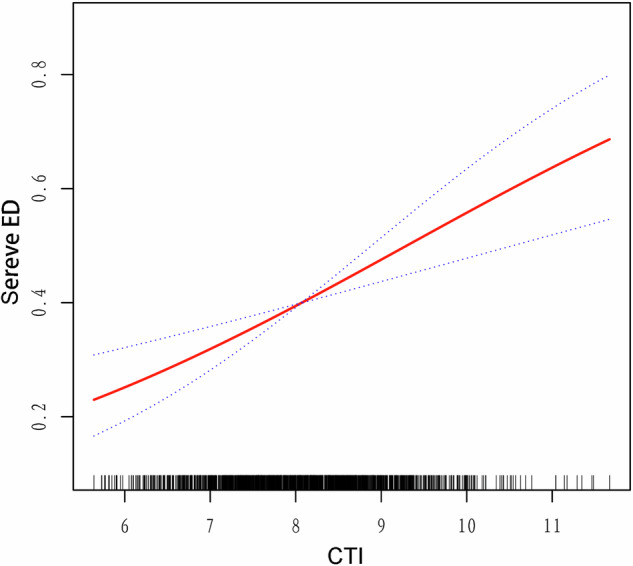
Density dose-response relationship between CTI with severe ED prevalence. The area between the upper and lower dashed lines is represented as 95% CI. The red line indicates that the positive linear association between CTI and ED is proven by generalized additive model. Adjusted for all confounders in model 2 included age, race, PIR, marital status, educational level, BMI, smoking status, alcohol intake, hypertension, DM, CVD, hypercholesterolemia, moderate physical activity, and vigorous physical activity.

**Fig. 5:**
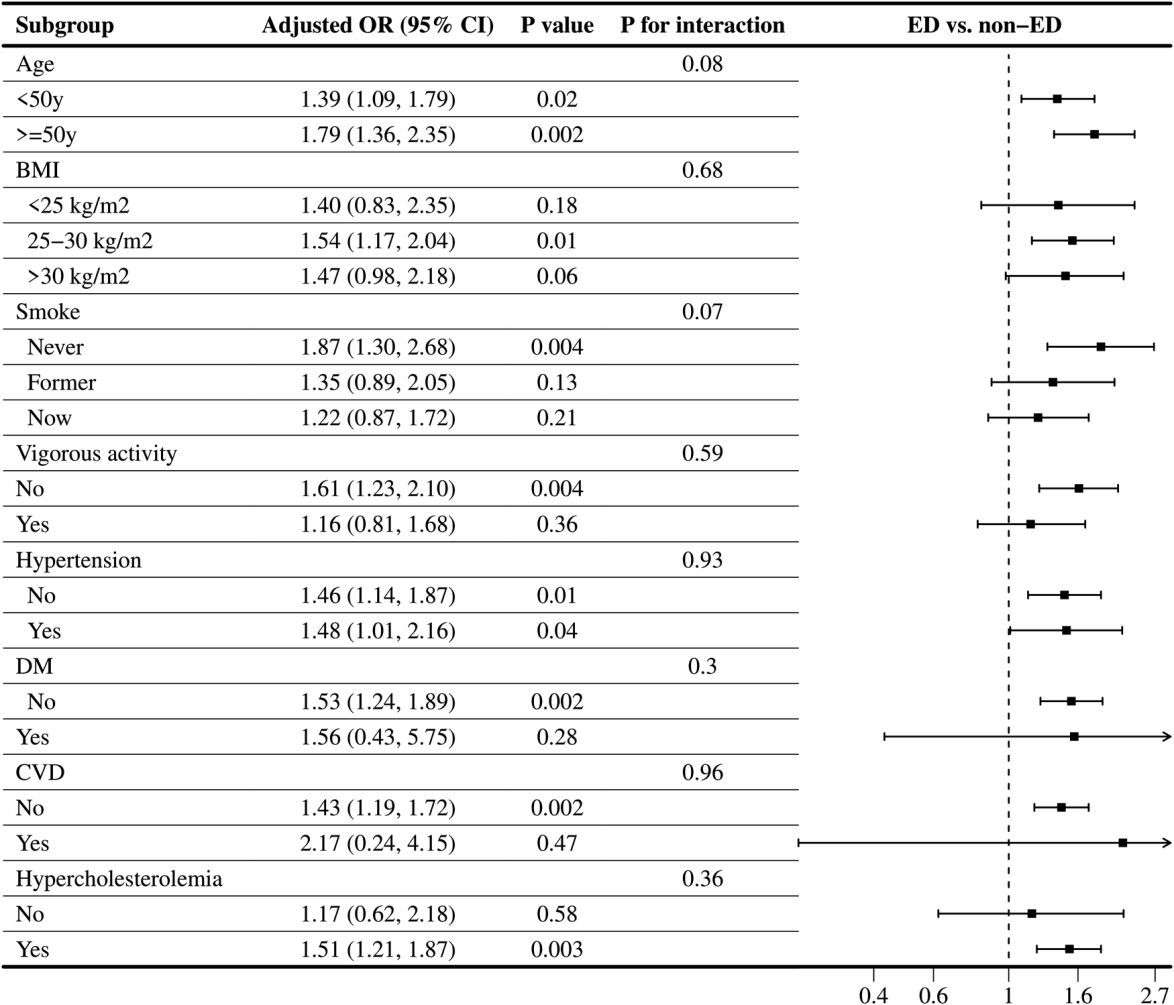
Subgroup analysis for the relationship between CTI and severe ED with CTI as continuous variable. All factors including age, BMI, smoking status, vigorous activity, and a history of hypertension, DM, CVD, and hypercholesterolemia had no impact on the independent positive association between CTI and severe ED. All subgroups were adjusted for age, race, PIR, marital status, educational level, BMI, smoking status, alcohol intake, hypertension, DM, CVD, hypercholesterolemia, moderate physical activity, and vigorous physical activity, except the stratification factor itself.

In the sensitivity analysis, participants who were classified as having more severe ED were those who reported being “never able to get and maintain an erection.” The significant relationship between the CTI and severe ED incidence persisted across all models, as detailed in Table 4. According to fully adjusted Model 2, each additional increase in the CTI was associated with a significant 44% rise in ED risk (OR = 1.44, 95% CI: 1.19–1.76; *P* = 0.004). After categorizing the CTI into four quartiles, logistic regression revealed a significant 1.39-fold increase in ED incidence in the highest CTI group compared to the lowest CTI group (OR = 2.39, 95% CI: 1.34–4.26; *P* = 0.01). Generalized additive modeling with smooth curve fitting consistently demonstrated a positive linear relationship between the CTI and severe ED (Fig. 4). Additionally, the subgroup analyses are presented in Table 5 and Fig. 5. Consistent with prior findings, the results indicated no significant impact on the positive correlation between the CTI and ED across all subgroups. Notably, we failed to perform subgroup analysis based on the presence of CVD with CTI as a categorical variable due to the lack of participants with CVD.

**Table 4:** Sensitivity analysis for the association between CTI with severe ED, weighted.

Exposure	Crude model		Adjusted model 1		Adjusted model 2	
	OR (95% CI)	*P*-value	OR (95% CI)	*P*-value	OR (95% CI)	*P*-value
CTI (continuous)	1.71 (1.48, 1.97)	<0.0001	1.55 (1.34, 1.79)	<0.0001	1.44 (1.19, 1.76)	0.004
CTI (Quartiles)						
Q1 (5.64–7.42)	Ref.		Ref.		Ref.	
Q2 (7.42–8.06)	1.72 (1.21, 2.44)	0.004	1.56 (1.08, 2.24)	0.02	1.53 (0.89, 2.63)	0.09
Q3 (8.06–8.65)	1.76 (1.22, 2.56)	0.004	1.50 (1.04, 2.18)	0.03	1.41 (0.75, 2.64)	0.21
Q4 (8.65–11.67)	3.66 (2.56, 5.23)	<0.0001	2.93 (2.01, 4.27)	<0.0001	2.39 (1.34, 4.26)	0.01
*P* for trend	<0.0001		<0.0001		0.006	

Statistical tests: Crude Model: no covariates were adjusted.

Model 1: age, race, PIR, marital status, and educational level were adjusted.

Model 2: Model 1 + BMI, smoking status, alcohol intake, hypertension, DM, CVD, hypercholesterolemia, moderate physical activity, and vigorous physical activity were adjusted.

*CTI* C-reactive protein-triglyceride glucose index, *ED* Erectile dysfunction, *OR* odds ratio, *CI* confidence interval, *Q1–Q4* quartile 1 to quartile 4, *CVD* cardiovascular disease, *DM* diabetes mellitus, *BMI* body mass index.

**Table 5:** Sensitivity subgroup analysis between CTI quartiles with severe ED in fully adjusted model 2 except the stratification factor itself, weighted.

Subgroup	Q1	Q2	Q3	Q4	*P* for trend	*P* for interaction
	Ref.	OR (95% CI)	OR (95% CI)	OR (95% CI)		
Age						0.15
<50 y	Ref.	1.45 (0.74, 2.85)	1.60 (0.73, 3.53)	2.09 (0.98, 4.47)	0.05	
≥50 y	Ref.	1.94 (0.71, 5.27)	1.47 (0.65, 3.31)	4.07 (2.04, 8.11)	0.003	
BMI						0.91
<25 kg/m^2^	Ref.	1.90 (0.74, 4.89)	1.51 (0.39, 5.84)	1.54 (0.39, 6.11)	0.32	
25~30 kg/m^2^	Ref.	1.66 (0.77, 3.57)	1.55 (0.61, 3.93)	2.85 (1.40, 5.81)	0.01	
>30 kg/m^2^	Ref.	0.87 (0.26, 2.94)	0.83 (0.21, 3.24)	1.66 (0.46, 6.01)	0.12	
Smoke						0.06
Never	Ref.	2.22 (1.05, 4.68)	2.68 (1.18, 6.10)	3.63 (1.52, 8.68)	0.01	
Former	Ref.	1.28 (0.55, 2.99)	0.54 (0.20, 1.45)	2.39 (0.83, 6.91)	0.13	
Now	Ref.	0.99 (0.39, 2.51)	1.32 (0.37, 4.69)	1.41 (0.50, 3.99)	0.38	
Vigorous activity						0.41
No	Ref.	1.32 (0.62, 2.79)	1.36 (0.50, 3.70)	2.85 (1.18, 6.88)	0.01	
Yes	Ref.	1.87 (0.80, 4.39)	1.47 (0.54, 4.03)	1.34 (0.41, 4.36)	0.59	
Hypertension						0.11
No	Ref.	1.20 (0.65, 2.22)	1.45 (0.73, 2.90)	2.07 (1.15, 3.73)	0.02	
Yes	Ref.	3.30 (1.04, 10.51)	1.76 (0.67, 4.62)	3.76 (1.33, 10.65)	0.05	
DM						0.30
No	Ref.	1.65 (0.96, 2.83)	1.41 (0.74, 2.71)	2.64 (1.51, 4.64)	0.004	
Yes	Ref.	0.14 (0.01, 1.38)	0.55 (0.07, 4.14)	0.70 (0.07, 6.55)	0.33	
CVD						-
No	Ref.	1.46 (0.87, 2.45)	1.37 (0.76, 2.47)	2.33 (1.38, 3.96)	0.004	
Yes	Ref.	-	-	-	-	
Hypercholesterolemia						0.34
No	Ref.	1.26 (0.41, 3.85)	1.19 (0.44, 3.22)	0.41 (0.06, 2.77)	0.80	
Yes	Ref.	1.83 (0.92, 3.64)	1.75 (0.87, 3.53)	3.04 (1.53, 6.02)	0.003	

Statistical tests: Model 2 controlling for age, race, PIR education, marital status, BMI, alcohol use, smoking, diabetes, hypertension, CVD, hypercholesterolemia, moderate physical activity, and vigorous physical activity were adjusted.

*CTI* C-reactive protein-triglyceride glucose index, *ED* Erectile dysfunction, *OR* odds ratio, *CI* confidence interval, *Q1–Q4* quartile 1 to quartile 4, *CVD* cardiovascular disease, *DM* diabetes mellitus, *BMI* body mass index, *PIR* poverty income ratio.

## Discussion

To the best of our knowledge, this is the first large-scale analysis to assess the association between CTI and ED incidence using a diverse sample of US men. Our findings suggest that there is a positive linear association between the CTI and the incidence of ED, highlighting this correlation in participants with more severe ED. The positive association persisted when the CTI was categorized into quartiles (Q1–Q4). Additionally, subgroup analyses showed that the relationship between the CTI and ED remained stable across all stratification variables, demonstrating a persistent positive correlation. Our study supports previous arguments that IR and chronic inflammation are the underlying mechanisms of ED. Hence, incorporating the CTI into clinical settings may assist in identifying individuals at a heightened risk of ED in the general population.

One of the causes of ED is the development of atherosclerosis in the penile vasculature [30, 31]. However, the primary mechanism underlying atherosclerotic blood vessel formation is related to inflammation rather than lipid infiltration [32]. Moreover, inflammation is a key factor in endothelial dysfunction and contributes significantly to the development and progression of atherosclerosis [33]. Normally, vascular endothelial cells possess anti-inflammatory properties. However, when the body’s equilibrium is disturbed, elevated oxidative stress and inflammation can trigger endothelial cell dysfunction [34]. Oxidative stress generates reactive oxygen species and promotes the formation of lipid peroxides, which leads to endothelial inflammation and subsequently accelerates endothelial dysfunction [35]. The onset and worsening of endothelial dysfunction are associated with increased levels of inflammatory markers and mediators, including CRP, intercellular and vascular cell adhesion molecules, fibrinogen, interleukin (IL) 1b, and IL-6 [36, 37]. CRP, a common inflammatory factor, may be an independent predictor of ED occurrence and severity [38–40]. CRP not only inhibits eNOS activity but also promotes the expression of P-selectin, chemokines, E-selectin, and vascular cell adhesion molecule-1 (VCAM-1), all of which can interfere with endothelial cells [41–43]. The resulting endothelial dysfunction exposes cells to proinflammatory cytokines, reducing NO production, ultimately inhibiting vasodilation, and leading to ED [44, 45]. In addition to this, CRP has the potential to impact erectile function by affecting testosterone levels in men [46].

ED is often strongly associated with endocrine-metabolic disorders, such as diabetes mellitus and metabolic syndrome (MetS), in which IR plays a crucial pathologic role [12, 47]. IR is an independent risk factor for ED [48, 49]. Many studies have proposed mechanisms for the relationship between IR and ED. IR increases oxidative stress and inflammatory cytokine production in endothelial cells [50], leading to excessive NO depletion in tissues exposed to free radicals, and the subsequent decrease in NO disrupts endothelial function [51, 52]. Endothelin-b receptors are associated with endothelial dysfunction, increased reactive oxygen species, and increased vasoconstriction in erectile tissue, whereas IR increased endothelin-b receptor expression in the vasculature of obese rats with IR [53]. This alteration in endothelial function negatively feeds back to further deterioration of insulin metabolism [54]. IR is a symptom of pre-diabetes and its progression to diabetes parallels the progression of endothelial dysfunction to atherosclerosis [55]. In addition, IR status elevates basal serum insulin levels, which in turn activates the sympathetic nervous system and increases atherosclerotic risk factors, all of which contribute to ED [56, 57]. IR also increases endothelin-1, a potent arterial and venous vasoconstrictor, in the cavernous tissue of the penis [58]. In the state of IR, the Leydig cells of the testes secrete less testosterone, which in turn leads to ED [59].

The homeostatic model assessment (HOMA-IR) uses insulin and glucose levels to determine IR and is an alternative to the gold standard glucose clamp [60]. However, insulin testing is difficult to popularize, which limits the use of HOMA-IR methods [61]. The TyG index, calculated from easily available and inexpensive fasting glucose and triglyceride indices [62, 63], has a significant positive correlation with the HOMA-IR [64] and has become an alternative to the HOMA-IR. The TyG index is an appropriate tool for diagnosing IR and, in some cases, is even superior to the HOMA-IR [54, 61, 65]. Currently, the TyG index is strongly associated with ED [19, 20].

A novel indicator, the CTI (which combines CRP and the TyG index to comprehensively assess the severity of inflammation and IR) is potentially valuable for predicting survival in cancer patients [25, 26]. As mentioned above, both CRP and the TyG index are strongly associated with ED. Hence, there may also be an association between the CTI and ED, and our findings confirm this hypothesis. The CTI serves as a straightforward and cost-effective metric that combines inflammation and IR measurements; it holds promising potential as a predictive tool for identifying individuals at risk of developing ED in the future.

Notwithstanding, it is essential to recognize certain limitations in our study when interpreting the findings. First and foremost, ED was defined based on a single self-reported question. Although validated as a surrogate, it is not the best option and can introduce significant recall bias or social desirability bias, which may interfere with the accuracy of the reported associations. Second, the results may only be generalizable to the specific demographics of the NHANES dataset, which predominantly covers the US population from 2001–2004. It is vital to remember that demographic traits have significantly shifted since then, potentially influencing the findings if the study were replicated and applied today. Moreover, inevitable sample selection bias exists, and only a small portion of the original sample met the predetermined inclusion criteria. Third, although we considered several important confounders, the presence of unmeasured/poorly measured variables (such as any psychological/psychiatric comorbidity or comorbid sexual dysfunction) could influence the observed associations. Finally, we cannot infer causality from this study, which is limited by the fundamental limitations of cross-sectional studies. As such, future studies should include more diverse populations and consider more potential covariates to elucidate the causal relationship between CTI and ED.

## Conclusion

Our study that used data from a representative sample of the US population revealed a consistent and positive association between CTI and the incidence of ED. The CTI comprehensively reflects the body’s levels of IR and chronic inflammation, which are pathogenic mechanisms in the development of ED. Thus, this indicator can be considered for clinical application to promote early prevention or intervention of ED. However, broader and deeper studies in the future are warranted to validate our findings.

## Supplementary information

aj-checklist.

## Data Availability

This study utilized publicly available data from the National Health and Nutrition Examination Survey (NHANES), and all details are accessible on the website (https://www.cdc.gov/nchs/nhanes). Further inquiries can be directed to the corresponding author.
